# Ramucirumab plus irinotecan / leucovorin / 5-FU versus ramucirumab plus paclitaxel in patients with advanced or metastatic adenocarcinoma of the stomach or gastroesophageal junction, who failed one prior line of palliative chemotherapy: the phase II/III RAMIRIS study (AIO-STO-0415)

**DOI:** 10.1186/s12885-023-11004-z

**Published:** 2023-06-19

**Authors:** Sylvie Lorenzen, Alix Schwarz, Claudia Pauligk, Eray Goekkurt, Gertraud Stocker, Jorge Riera Knorrenschild, Gerald Illerhaus, Tobias Dechow, Markus Moehler, Jean-Charles Moulin, Daniel Pink, Michael Stahl, Marina Schaaf, Thorsten Oliver Goetze, Salah-Eddin Al-Batran

**Affiliations:** 1 Klinikum rechts der Isar, Technische Universität München, III. Medizinische Klinik und Poliklinik, München, Germany; 2 Institut für Klinische Krebsforschung IKF GmbH am Krankenhaus Nordwest, Frankfurt/Main, Germany; 3 Hämatologisch-Onkologische Praxis Eppendorf (HOPE), und Universitäres Cancer Center Hamburg (UCCH), Hamburg, Germany; 4 Universitäres Krebszentrum Leipzig (UCCL), Klinik und Poliklinik für Onkologie, Gastroenterologie, Hepatologie, Pneumologie und Infektiologie – Bereich Onkologie Leipzig, Leipzig, Germany; 5 Universitätsklinikum Marburg, Klinik für Innere Medizin, Hämatologie, Onkologie und Immunologie, Marburg, Germany; 6 Klinikum Stuttgart, Klinik für Hämatologie, Onkologie und Palliativmedizin, Stuttgart, Germany; 7 Studienzentrum Onkologie Ravensburg, Ravensburg, Germany; 8 I. Department of Internal Medicine, University Cancer Center Mainz, Mainz, Germany; 9 Ortenau Klinikum Lahr, Medizinische Klinik, Sektion Hämatologie/Onkologie, Lahr, Germany; 10 Klinik und Poliklinik für Innere Medizin C, Hämatologie und Onkologie, Transplantationszentrum, Palliativmedizin, Universität Greifswald, Greifswald, and Klinik für Hämatologie, Onkologie und Palliativmedizin, Sarkomzentrum Berlin-Brandenburg, HELIOS Klinikum Bad-Saarow, Bad Saarow, Germany; 11 Evang. Kliniken Essen-Mitte, Klinik für Internistische Onkologie und Hämatologie, Essen, Germany; 12 University Cancer Center Frankfurt, Krankenhaus Nordwest, Institut für Klinisch-Onkologische Forschung, Frankfurt/Main, Germany

**Keywords:** Metastatic esophagogastric adenocarcinoma, Ramucirumab, Second line treatment, Paclitaxel, FOLFIRI

## Abstract

**Background:**

Paclitaxel in combination with ramucirumab is the standard of care second-line therapy in gastro-esophageal adenocarcinoma (GEA). As the number of taxane pretreated patients in the perioperative or first-line setting is increasing, it is unknown whether these patients benefit from re-applying a taxane in using the combination of paclitaxel and ramucirumab. Furthermore, the rates of neurotoxicity with first-line FOLFOX or FLOT range from 30%–70%, making second-line taxane‐containing therapy less suitable to a meaningful portion of patients. This patient group is likely to benefit from a taxane-free second-line chemotherapy regimen, such as FOLFIRI and ramucirumab (FOLFIRI-Ram). Therefore, the RAMIRIS phase III trial evaluates the effects of the regimen of FOLFIRI-Ram in the second-line treatment after a taxane-based chemotherapy in patients with advanced GEA.

**Methods:**

The RAMIRIS trial is a randomized, open-label, multicenter phase II/III study comparing treatment of FOLFIRI-Ram (arm A) with paclitaxel and ramucirumab (arm B). The Phase II is already closed with 111 enrolled patients. In the phase III, 318 taxane-pretreated patients with advanced GEA will be recruited and randomized 1:1 to FOLFIRI (5-FU 2400 mg/m^2^ over 46 h i.v., irinotecan 180 mg/m^2^ i.v.; 5-FU 400 mg/m^2^ bolus; leucovorin 400 mg/m^2^ i.v.; on day 1 and 15, q28) with ramucirumab 8 mg/kg every two weeks (Arm A) or paclitaxel 80 mg/m^2^ (days 1, 8, 15, q28) with ramucirumab 8 mg/kg every two weeks (Arm B). The primary endpoints are overall survival (OS) and objective overall response rate (ORR). Secondary endpoints are progression-free survival (PFS), disease control rate and safety and quality of life as assessed by EORTC-QLQ-C30 questionnaire.

**Discussion:**

The already completed RAMIRIS phase II demonstrated feasibility and efficacy of FOLFIRI-Ram. Especially docetaxel-pretreated patients seemed to markedly benefit from FOLFIRI-Ram, with favorable response- and PFS rates and lower toxicity. This offers a rationale for the phase III trial. If the RAMIRIS III trial transfers and confirms the results, they will affect the current treatment guidelines, recommending the combination therapy of FOLFIRI-Ram for taxane-pretreated patients with advanced GEA.

**Trial registration:**

NCT03081143 Date of registration: 13.11.2015

## Background

Gastro-esophageal adenocarcinoma (GEA) is the fifth most common cancer worldwide, causing over one million new cases (1,033,701) each year and is the third-leading cause of cancer death (about 782,685 deaths and 8.2% of total in 2018) [1].

At initial diagnosis more than two thirds of patients are not suitable for curative treatment, because they have an advanced stage GEA [1, 2]. Despite improved molecular analysis, the actual worldwide standard for advanced GEA is a platinum‐ and fluoropyrimidine (5 FU)‐based regimen [3, 4]. Trastuzumab can be given additional in HER-2 positive GEA [5]. Triplet- regimens such as FLOT have only a marginal benefit with the addition of docetaxel versus doublet- regimens but an increase in toxicities [6–8]. The median overall survival (mOS) for patients with advanced GEA receiving first-line-therapy is around one year [9–13].

In the long-term, nearly all patients fail to first line-line therapy and suffer from disease progression. Only 42%-54% of those patients receive another treatment regimen [14, 15]. Second-line therapy has an inferior outcome compared to first-line therapy and studies investigating this further are still lacking.

Ramucirumab is an accepted second line therapy in advanced GEA either as monotherapy or in combination with paclitaxel [16, 17]. Other VEGF(R)-antibodies such as Bevacizumab in combination with chemotherapy, could not enhance OS significantly. Although a clinically meaningful improvement of OS of 2 months could be demonstrated in the AVAGAST trial [18].

In the second-line setting Irinotecan as a monotherapy or in combination with 5-FU/Folinic Acid (FOLFIRI) is a safe and efficient regimen and has shown a significant improvement of OS compared to BSC for patients with progressive GEA [19–21]. The FOLFIRI regimen could improve overall survival to 9.1 months, and patients achieved an overall response rate (ORR) of 18% and a progression-free survival (PFS) of 3.2 months with acceptable tolerability in an Asian patient population [22].

With regards to toxicity, additional support for the safety and efficacy of FOLFIRI in combination with ramucirumab (FOLFIRI‐Ram) was established in the RAISE trial in second-line advanced colorectal cancer after progression on 5-FU/Folinic acid and Oxaliplatin (FOLFOX) with bevacizumab [23].

### Study rationale

Current treatment algorithms recommend a taxane- containing triplet regimen for selected patients as first-line therapy for advanced GEA [4]. For patients with a locally advanced and potentially operable GEA the perioperative chemotherapy regimen FLOT (5-FU/Folinic acid, Oxaliplatin, Docetaxel) is the current standard treatment with an improvement of 15 months in OS vs. ECX/F (Epirubicin, Cisplatin, capecitabine/ fluorouracil), as shown in the FLOT4 trial [24]. An alternative option is a neoadjuvant radio-chemotherapy regimen according to the CROSS – trial (41 Gy, Carboplatin AUC 2 and Paclitaxel 50 mg/m^2^) for patients with gastroesophageal junction tumors [25]. An improvement of the 3-year relapse-free survival by over 15% was shown by extending the adjuvant chemotherapy of S-1 by docetaxel for resected patients with stage III GEA in the Japanese JACCRO GC-07 trial [26].

Recurrence rates for initially curative treated patients remain high, ranging between 36% to about 70% for patients who received a taxane containing perioperative regimen [27–29]. This leads to a large group of patients who are taxane pre-treated. For those patients it is still unclear, whether they benefit from second-line treatment reapplying a taxane in the combined regimen with ramucirumab and paclitaxel, as patients with taxane-pretreatment were excluded from the RAINBOW trial. Ramucirumab as a monotherapy or in combination with paclitaxel as a second-line treatment is a proven option, as stated above. Given that there are no clear guidelines, many oncologists would argue for a regimen containing irinotecan as a second-line treatment. Therefore, at the time of the RAMIRIS phase II trial initiation, there was a great need to examine an irinotecan-based regimen together with ramucirumab. Since that, the need even increased to answer the question about the optimal combination partner for ramucirumab in patients who had received a taxane. The subgroup analysis of the phase II part of the RAMIRIS trial provided another rationale for the phase III part. It showed a trend for a risk reduction for death for taxane retreated patients in the FOLFIRI group with a mOS of 7,5 months (95% CI 5.9–12.94) compared to 6,6 months (95% CI 0.47–1.43) for the paclitaxel group [30].

As a significant number of patients with GEA suffer from neurotoxic side-effects after first-line treatment with FOLFOX (30–70%) a second line treatment containing a taxane is less reasonable for this group [31, 32]. In addition, first-line treatments containing a taxane or early recurrence after FLOT therapy in the curative setting makes a second-line taxan-free option highly needed [11, 12, 33, 34].

The safety analysis of the phase II RAMIRIS trial did not reveal any unexpected safety issues. The results of the safety interim analysis provide further support for the safety and efficacy of second-line FOLFIRI-Ram in this patient group [35].

Therefore, the Arbeitsgemeinschaft Internistische Onkologie (AIO) investigators implemented a phase III portion of the RAMIRIS phase II trial, which is currently ongoing (NCT03081143). The phase III portion will not utilize the patients enrolled into the phase II portion.

The phase III portion of the RAMIRIS trial will evaluate whether the combination of FOLFIRI-Ram (investigational arm A) is superior in terms of OS and ORR compared to the standard treatment of ramucirumab plus paclitaxel (control arm B) in patients who had received a taxane (docetaxel or paclitaxel). This might lead to a new standard of care in this particular group of patients by changing the national and international guidelines.

## Methods / Design

The RAMIRIS trial is a randomized, open-label, multicenter phase II/III study comparing treatment of FOLFIRI-Ram (arm A) with paclitaxel and ramucirumab (arm B) in patients with GEA after prior taxane containing therapy. The study will evaluate the safety and efficacy of the treatment regimens including quality of life.

**Fig. 1:**
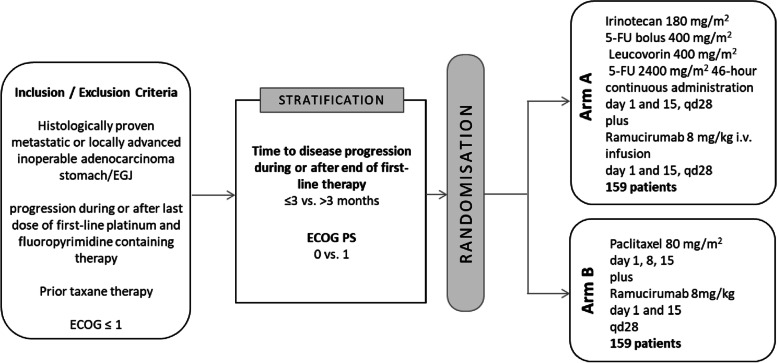
Study design of the RAMIRIS Phase III Trial

A total number of 318 patients will be recruited, from which 159 patients will be randomized in arm A (FOLFIRI-Ram) and 159 will be treated with paclitaxel and ramucirumab (arm B) (Fig. 1). The randomization will occur in a 1:1 ratio with stratification by disease progression (≤ 3 months vs. > 3 months during or after first-line therapy) and ECOG PS (0 vs. 1). After the randomization the study patients will enter the study treatment period which will last for a maximum of 1 year. Patients who benefit from the study therapy beyond the study period will continue treatment and will be included in the follow-up.

The protocol has been approved by the individual institutional ethics committees. All patients provided written informed consent prior to study participation.

The primary endpoint of the phase III trial is to compare OS in patients with locally advanced and inoperable or metastatic GEA receiving FOLFIRI-Ram versus paclitaxel and ramucirumab as second-line therapy and who failed prior taxane-containing therapy in the intent to treat population (ITT).

Secondary endpoints are to compare the disease control Rate and the PFS as well as the quality of life between the two treatment arms. The safety and tolerability of ramucirumab and FOLFIRI or paclitaxel are evaluated by monitoring the incidence, frequency and severity of adverse events (AE) according to NCI-CTCAE V 4.03 [36].

### Participants

Eligible patients need to fulfill all of the following criteria:

1. Signed written informed consent
2. Women or men ≥ 18 years of age; Patients in reproductive age must be willing to use adequate contraception during the study and for 3 months after the end of ramucirumab treatment. Women of childbearing age need to have a negative pregnancy test within 7 days before study start. Patients will be enrolled gender-independently.
3. Proven histology of gastric adenocarcinoma including adenocarcinoma of the esophagogastric junction
4. Metastatic or locally advanced, inoperable disease
5. Radiological or clinical disease progression during or after the last dose of a first-line platinum, fluoropyrimidine-containing therapy. Patients must also have received a taxane with the first-line and/or during their adjuvant or neoadjuvant therapy in a curative setting. Neoadjuvant/adjuvant platinum containing therapy is permitted and is counted as first-line therapy if progression occurs within 12 months after completion of the treatment. The therapy is not considered as a treatment line, if progression or recurrence occurs 12 months after end of last treatment. In case of different prior treatments, they can be considered as one therapy line, if they were administrated as a continuous or alternating therapy.^1^
6. Measurable or non-measurable but evaluable disease
7. Eastern Cooperative Oncology Group Status must be less than or equal to 0–1
8. Expectancy of life must be less than or equal to 12 weeks
9. Reasonable hematological, hepatic and renal functions:
  a Absolute neutrophil count (ANC) ≥ 1.5 x 109/L
  b Platelets ≥ 100 x 109/L
  c Hemoglobin ≥9 g/dL (5.58 mmol/L
  d Total bilirubin ≤ 1.5 UNL
  e AST (SGOT) and ALT (SGPT) ≤ 2.5 x UNL without liver metastases, or ≤ 5 x UNL in case of liver metastases; AP ≤ 5 x UNL
  f Serum creatinine ≤ 1.5 x ULN, or creatinine clearance ≥40 mL/minute
  g Urinary protein ≤1+ on dipstick or routine urinalysis; if urine dipstick or routine urine analysis is ≥2+ of urinary protein, patients need a 24-hour urine collection and protein must be below <1000 mg in 24 hours
  h An adequate coagulation function measured by the International Normalized Ratio ≤ 1.5 and the partial thromboplastin time ≤ 5 seconds above the ULN (unless receiving anticoagulation therapy). Patients receiving warfarin/ phenprocoumon must be changed to low molecular weight heparin and need to show a stable coagulation profile before start of first dose of therapy
10. Capability to follow scheduled assessments and to dope with side effects.

### Study treatments

#### Arm A - Experimental Treatment consists of:

- Ramucirumab 8 mg/kg intravenously on day 1, 15; q28
- FOLFIRI (intravenously: Irinotecan 180 mg/m^2^, bolus of 5-FU 400 mg/m^2^, leucovorin^2^ 400 mg/m^2^, 5-FU 2400 mg/m^2^ 46 h-continous administration) on day 1,15; q28

#### Arm B - Standard Treatment consists of:

- Ramucirumab 8 mg/kg intravenously on day 1, 15; q28
- Paclitaxel 80 mg/m^2^ intravenously on day 1, 8, 15; q28

Each cycle will be repeated after 28 days (from day 1) for a maximum of 1 year.

### Assessment

Tumor assessment for study inclusion will be performed within 4 weeks prior to the first dose of study therapy and every eight weeks while treatment. The assessment will be performed by CT scan or MRI from chest to pelvis and all other sites of metastases and should continue until progression. Patients who discontinue trial therapy prior to disease progression should continue to have tumor assessments as per protocol schedule until progression. Tumor assessments are evaluated in accordance with to RECIST 1.1 [37].

### Follow up

After discontinuation of study medication patients will be followed up for at least 1 year, every 2 months (except for the first follow up visit, which will be after 30 days due to safety reasons). Tumor assessments per CT scan or MRI will be performed every 8 weeks or until disease progression according to clinical routine.

#### Statistical methods and data analysis

The intention of the RAMIRIS Phase III trial is to show a superior therapeutic efficacy of the experimental regimen FOLFIRI-Ram compared to the combination of paclitaxel and ramucirumab in patients pretreated with a taxane. Accordingly, the research hypothesis of the study is one-sided. The primary endpoints are OS and ORR according to RECIST. OS is defined as the time from randomization to death from any cause. Further the aim is to compare the ORR in the two groups. ORR is defined as the percentage of patients with complete or partial remission as their best overall response based on Response evaluation criteria in solid tumors 1.1. (RECIST) [38].

A Bonferroni type adjustment of the α error level, due to multiple testing, will be applied. The OS and PFS will be estimated by using the Kaplan–Meier method and logrank test. Hazard ratios will be obtained from corresponding Cox proportional hazard models. Chi^2^ test will be used to compare the treatment groups regarding their ORR. Patients´ data from the phase II portion of RAMIRIS are not included in the primary analysis of the phase III portion. Patients who drop out of the study or who are lost to follow-up are censored at their date of EOT respectively at the last date known.

All parameters (except for the co-primary endpoints) will be measured in an explorative or descriptive way. All p values will be two-sided if not explicitly mentioned (co- primary endpoints of phase III). The suitability of the methods will be reevaluated after the data has been received. If required, the statistical method will be adapted accordingly, with critical discussion.

OS, ORR, rate of toxicity and other event rates are estimated at pre-defined time points, confidence intervals will be provided. For data analysis Fisher ´s exact test, chi^2^ test or Mantel–Haenszel test or trend test according to Cochran/Armitage, will be used. Multivariate analyses will be executed by Cox analyses [37].

No formal interim analyses on efficacy or futility are planned for the phase III portion.

### Sample size estimation

Looking at results of the RadPAC Trial [39] and on results of the interim analyses of the standard arm of the phase II RAMIRIS trial, the median OS in the standard arm is approximately 6 months. An increase to 8.6 months (hazard ratio of HR = 0.70) in the experimental arm appears to be meaningful. In total *n* = 264 events have to be observed, based on an α error of 0.020 (one-sided) and a high confidence level (power = 80%) to be able to detect such an improvement in the experimental arm. Under the following assumptions 318 eligible patients are needed to reach the needed number of events. The recruitment period is 18 months, 1 year as minimum follow-up time after last recruited patient, exponential distribution of the survival curves, a 5% drop-out rate.

On the other hand, only 298 patients are aquired to ensure 80% power to detect an improvement from 10 to 25% for the co-primary endpoint ORR based on an one-sided α error of 0.005. Therefore, 318 patients will be randomized.

## Discussion

A high number of first-line taxane-treated patients with GEA relapse and require a second-line treatment. However, data is scarce, whether they benefit more from a re-exposure to a taxane-containing therapy, such as the standard regimen paclitaxel and ramucirumab, than from a taxane-free regimen. Therefore, the Phase III RAMIRIS trial will prospectively provide evidence on the safety and efficacy of second-line FOLFIRI‐Ram after progression on first-line platinum/5-FU in patients pretreated with a taxane.

Clinical data shows, that second-line treatment with FOLFIRI-Ram is safe and shows no additional neurotoxicity. In the evaluation of the RAMIRIS phase II portion the feasibility of FOLFIRI-Ram could be confirmed: Grade 3 or higher AEs reported were mainly neutropenia (20% in die experimental group vs. 22% in the standard group), diarrhea (8% in die experimental group vs. 3% in the standard group fatigue) and.fatigue (6% in die experimental group, no fatigue was reported in the standard group). Serious treatment-related AEs were observed in 14% of patients in the experimental group and 23% of patients in the standard group [30, 40].

Studies show that the combination of FOLFIRI-Ram or Irinotecan and ramucirumab compares favorably to the standard regimen paclitaxel and ramucirumab [41–44]. Vogl et al. showed promising results in a retrospective analysis for the combination of FOLFIRI-Ram as a second-line treatment compared to paclitaxel and ramucirumab. 56 patients with GEA were treated with second-line or beyond second-line treatment with either paclitaxel-Ram (*n* = 38) or FOLFIRI-Ram (*n* = 16). FOLFIRI-Ram was given to patients with an early relapse after perioperative chemotherapy or ineligible for paclitaxel. The mPFS in FOLFIRI-Ram group was significantly longer than in the paclitaxel-Ram group (5.9 vs. 2.9 months), with an ORR of 23% (partial response) vs. 9.4% [45].

The study was designed and initiated in 2015. During this time, no patients with GEA were treated with immunotherapy as standard of care. Therefore, pre-treatment with immunotherapy was not explicitly mentioned in the eligibility criteria. Since immunotherapy is now standard of care for selected patients according to the CPS (combined positive score), patients pretreated with immunotherapy can be included. There are even signals from retrospective analysis that immunotherapy pretreated patients have an improved survival when subsequently treated with the combination of Ramucirumab plus chemotherapy versus chemotherapy alone due to a synergistic effect of immunotherapy and anti VEGF-receptor therapy [46, 47].

The outcomes of the retrospective study evaluating FOLFIRI-Ram as a second-line treatment for patients with GEA by Klempner et al. and the phase III trial investigating ramucirumab and irinotecan in third-line treatment or beyond for GEA patients by Sakai et al. resulted already in the inclusion of FOLFIRI-Ram or irinotecan-Ram in the NCCN Clinical Practice Guidelines as a second-line treatment for GEA [48, 49].

In summary published data strongly supports the rationale for expansion to phase III of the RAMIRIS trial. If the results can be confirmed they will affect the current treatment guidelines, recommending the combination therapy of FOLFIRI-Ram for taxane-pretreated patients with advanced GEA.

## Data Availability

Institut für Klinische Krebsforschung IKF GmbH am Krankenhaus Nordwest, Steinbacher Hohl 2–26, 60,488 Frankfurt am Main, Germany, www.ikf-khnw.de.

## Abbreviations

5-FU: 5-Fluorouracil
AE: Adverse Event
AIO: Arbeitsgemeinschaft internistische Onkologie
BSC: Best Supportive Care
CR: Complete Response
DCR: Disease Control Rate
ECX/F: Epirubicin, Cisplatin, capecitabine/ fluorouracil
EOT: End of Treatment
FOLFIRI: 5-FU/Folinic acid, Irinotecan
FOLFOX: 5-FU/Folinic acid, Oxaliplatin
FLOT: 5-FU/Folinic acid, Oxaliplatin, Docetaxel
GEA: Gastro-Esophageal Adenocarcinoma
mOS: Median overall survival
ORR: Overall Response Rate
OS: Overall Survival
mPFS: Median Progression-free survival
PFS: Progression-free survival
RECIST: Response Evaluation Criteria in Solid Tumors
VEGF: Vascular endothelial growth factor

## Footnotes

1 The Inclusion criteria differs from the phase II part of the RAMIRIS trial, since in phase III part it is mandatory, that the patient has already received a taxane in a previous regimen.

2 Leucovorin can be exchanged for sodium folinate in accordance with local guidelines.
